# Community Engagement Studio as a Strategic Tool for Tailoring a Survey on Dental Care Access Among Adolescents

**DOI:** 10.1111/jphd.12682

**Published:** 2025-06-03

**Authors:** Rubelisa C. G. de Oliveira, Hassan Khalid, Grace McKenzie, Laurene Tumiel‐Berhalter, Jessica S. Kruger

**Affiliations:** ^1^ Department of Periodontics and Endodontics, School of Dental Medicine University at Buffalo Buffalo New York USA; ^2^ School of Public Health and Health Professions University at Buffalo Buffalo New York USA; ^3^ Clinical and Translational Science Institute University at Buffalo Buffalo New York USA; ^4^ Department of Family Medicine, Jacobs School of Medicine and Biomedical Sciences University at Buffalo Buffalo New York USA; ^5^ Community and Stakeholder Engagement Research Module, Clinical and Translational Science Institute University at Buffalo Buffalo New York USA; ^6^ Department of Community Health and Health Behavior, School of Public Health and Health Professions University at Buffalo Buffalo New York USA

**Keywords:** community dentistry, community health, dental health survey, health services, social determinants of health

## Abstract

**Objectives:**

The Community Engagement (CE) Studio aimed to provide feedback on a survey related to dental care access among adolescents. This approach allowed the research team to ensure that the survey integrated both the investigator's perspective and direct input from the community, ensuring the survey was clear, relevant, and accessible to its intended audience.

**Methods:**

The CE Studio is an efficient consultative model for researchers to gain community input on the development, implementation, or dissemination of a research project. The CE Studio was conducted to inform the design of a survey on dental care access, based on the social determinants of health. Parents or caregivers of adolescents with limited access to dental care living in an underresourced area in Buffalo, New York, participated in the session, providing valuable insights and recommendations for the survey's construction.

**Results:**

The CE Studio participants provided valuable feedback, including suggestions for motivating participation in the survey, changes in demographic questions, and the survey layout. Furthermore, the participants provided new insights into their dental access experiences and barriers to care, leading to additional questions for the survey. Finally, the participants suggested strategies to streamline survey instructions, provided their preferred wording for survey items and recommended how questions should be displayed to future research participants. Overall, 130 edits were made to the original survey, yielding a clearer, more representative, and accessible format.

**Conclusion:**

CE Studios can positively impact the design of recruitment materials, survey materials, and question structures in community‐focused dental health research.

## 1 Introduction

Access to dental care services is important for oral health promotion, education, and early disease diagnosis and management. People with better access to dental services tend to have better oral health outcomes, leading to fewer school or work absences due to unmet dental needs, such as untreated caries and toothache [1, 2, 3, 4]. Despite the recognized importance of oral health as a key component of overall well‐being, populations experiencing health disparities often face significant barriers to access appropriate dental services [5, 6]. Consequently, there are considerable disparities in tooth loss [7, 8], tooth decay [9, 10, 11], and oral health‐related quality of life [12], which are even more pronounced in adolescents.

In recent decades, the oral health of younger children has improved; however, the same progress has not been observed in adolescents [11]. The prevalence of caries in children aged 2–5 years decreased from 28% in 1999–2004 to 23% in 2011–2016, while it dropped from 21% to 17% in the same period among children aged 6–11 years. In contrast, adolescents aged 12–19 years continued to experience more than double the prevalence of untreated tooth decay (59%), representing a minimal change from 57% in 1999–2004. Additionally, the Healthy People 2030 report indicated a decrease in dental service utilization among low‐income youths, suggesting it would be difficult to reach the utilization target of 79.9% [13]. These factors emphasize the urgent need to address the oral and overall health needs of adolescents, a group often overlooked in research and healthcare strategies, despite the critical role adolescence plays in shaping long‐term health behaviors [14, 15].

While there are several instruments that assess the social and structural determinants of health, including oral health‐related topics, such as oral health literacy [16, 17, 18, 19], satisfaction with dental care [20], anxiety related to dental visits [21], oral health knowledge and behavior [22, 23], beliefs [24, 25], and provider empathy [26, 27], there is a notable lack of instruments specifically designed to address health disparities related to constructs of access to dental care. Table 1 provides a selection of currently validated instruments related to access to dental care and social determinants of health, but none have comprehensively examined the complex and multifaceted factors influencing access to dental care among adolescents who may experience health disparities.

**TABLE 1:** Validated instruments assembled to assess dental care access in different levels of influence.

Social determinant of health domain(s)^a^	Level(s) of influence^b^	Main topic	Instrument
Education	Intrapersonal and interpersonal	Health literacy	Oral Health Literacy Instrument (OHLI) [16]
Health care system	Intrapersonal, interpersonal, and structural	Accessibility and availability	Dental Satisfaction Questionnaire (DSQ) [20], Dental Care Survey Medicaid Managed Care Members [28], and Consumer Assessment of Healthcare Providers and Systems (CAHPS) [27]
Health behavior	Intrapersonal and interpersonal	Behavior during a dental visit	Modified Dental Anxiety Scale (MDAS) [21]
Community and social support	Interpersonal and structural	Provider's empathy	Dental Care Survey Medicaid Managed Care Members [28], Consultation and Relational Empathy (CARE) Measure [26], and Consumer Assessment of Healthcare Providers and Systems (CAHPS) [27]
Economic stability and built environment	Structural	Dental care access and barriers	Dental Care Survey Medicaid Managed Care Members [28]

^a^ The CDC refers to extensive lists of SDOH, which encompass five key domains and their connection with health and wellbeing: economic stability, education, health and health care, neighborhood and built environment, and social and community context [29].

^b^ Social ecological models for health promotion address factors in different levels of influence: intrapersonal, interpersonal, organizational/structural, community, and public policy [30].

This gap is concerning, especially given the consistent decrease in the proportion of low‐income youths who make annual preventive dental visits [13]. There is a need for a more holistic instrument that addresses multiple factors that influence decision‐making processes related to dental services for populations experiencing health disparities. We hypothesized that consulting parents or caregivers from these populations would help our research team develop a more comprehensive survey design, specifically focused on dental care access among adolescents. However, incorporating the community in research has proven to be challenging due to limited access and insufficient time and resources to engage the community effectively [31].

To address these challenges, the Meharry‐Vanderbilt Community‐Engaged Research Core developed the CE Studio in 2009, a one‐time consultative model, to gain valuable feedback from community members throughout the research process [32]. This approach has been useful, particularly in the early stages of project development. It helps researchers overcome barriers that might impact study feasibility, such as trust, bidirectional communication, power differences, scheduling conflicts, equitable recruitment, and compensation [33, 34, 35, 36, 37, 38, 39, 40].

Recognizing the value of the CE Studio, especially in considering the influence of social and structural factors on health, we applied this model to guide the construction of a survey for an upcoming study. By engaging with community members, we ensured that the instrument was clear, relevant, and accessible to its intended audience. Additionally, this approach helped our research team in tailoring the survey to investigate access to dental care among adolescents, with a particular focus on those facing health disparities, while remaining relevant to the broader adolescent population.

This is the first article to describe the use of the CE Studio as an important step in the development of an instrument for community and preventive dentistry research. Through this innovative approach, we hope to contribute to more effective and equitable solutions for improving access to dental care for families with health disparities.

## 2 Methods

### 2.1 Community Engagement Studio

CE Studios are one‐time consultative meetings in which community members provide direct feedback to research teams, ensuring that the research process is optimized for relevance, success, and impact [41]. These sessions allow researchers to gather critical insights that help align the study with community needs and perspectives. Many medical research institutions nationwide have established CE Cores, offering researchers the opportunity to conduct CE Studios, which are community consultations rather than research. The CE Core is responsible for all logistical aspects, including advertising, recruitment, organization, facilitation, recording, transcription, and participant compensation.

Institutional Review Board (IRB) approval is not required for CE Studio sessions. However, the associated research project must be approved. Therefore, the survey draft discussed in the CE Studio received IRB approval as part of a major project to identify barriers to accessing dental care among adolescents (STUDY00007027).

Our initial survey on access to dental care was informed by the five social determinants of health (SDoH) domains [42] and McLeroy's multiple levels of influence [30]. We identified validated instruments that addressed the SDoH domains across different levels of influence (Table 1), which were then used to select the questions included in our draft for discussion with the CE Studio participants, often called “community experts.”

The survey considered several topics including demographics, oral health status, access to care, barriers to care, oral health literacy, dental care satisfaction, and dental anxiety. All questions were drawn from established surveys, such as the Oral Health Literacy Instrument (OHLI) [16], the Dental Satisfaction Questionnaire (DSQ) [20], the Dental Care Survey for Medicaid Managed Care Members [28], the Consumer Assessment of Healthcare Providers and Systems (CAHPS) [27], and the Modified Dental Anxiety Scale [21]. The draft survey was reviewed and discussed by researchers from various fields and with different levels of experience, including public health, medical schools, and dental schools. This ensured that the draft survey was adequately prepared to receive feedback from the CE Studio participants.

### 2.2 Recruitment of Community Experts

**FIGURE 1:**
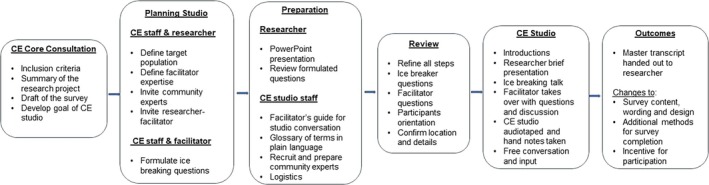
Workflow and step‐by‐step description of the Community Engagement (CE) Studio. [Color figure can be viewed at wileyonlinelibrary.com]

The CE Studio approach, adapted from the protocol established by Vanderbilt University, aimed to provide feedback on the draft survey related to dental care access among adolescents before its use in an upcoming study. Figure 1 shows the workflow of the CE Studio model.

The Buffalo Research Registry (BRR), a database of people interested in participating in research, was used to invite participants to the CE Studio. The BRR had about 7000 people enrolled at the time this CE Studio was conducted. A CE Studio flyer was sent to BRR members who self‐identified as non‐White, lived in underresourced areas in Buffalo, were interested in studies about children, and had an active email address (*N* = 474). Ten individuals expressed an interest in participating. Additionally, the flyer was emailed to 240 community contacts representing community‐based services, who were asked to share the flyer with clients. Two additional participants were recruited using this method.

These 12 participants were contacted by phone to confirm if they were parents or caregivers of adolescents living in an underresourced area in Buffalo and to collect their demographic information. During this phone call, a time was arranged for a subsequent telephone orientation, which included an overview of the CE Studio model, discussion of participants' expectations, and the collection of information for processing a $50 direct debit card payment as compensation. Orientation is critically important for potential CE Studio participants to know exactly what to expect, know the questions being asked, and make a final decision on whether to participate. After the orientation, participants received an email containing the CE Studio questions, draft survey, researchers' slide presentations, and a glossary of key terms in plain language. The University at Buffalo (UB) CE Core team also worked with the investigators to prepare short presentations and to develop questions for the CE Studio.

The CE Studio was facilitated by a community‐based researcher who collaborated with a local community center to provide free dental care to populations with health disparities living in an underresourced area in Buffalo. With her knowledge and commitment to the community, she was a natural fit as a facilitator for the presentations and conversations in the CE Studio.

### 2.3 Community Engagement Studio Process

The CE Studio was held in person at the Delavan Grider Community Center, a resource hub on the east side of Buffalo, which is a diverse, low‐income community. All 12 participants were non‐white and lived in underresourced areas in Buffalo. Following the protocol (Figure 1), the researcher conducted a 10‐min PowerPoint presentation in plain language on the research and perspective study. A roundtable conversation started with sharing lived experiences as an icebreaker, followed by questions about dental care. After the participants became comfortable with each other and the topics, questions about the research study with critiques of the survey, methodology, and recruitment were thoroughly discussed for 2 h.

The session was audiotaped using Zoom, and notes were taken on a laptop to assist with transcription. In addition, handwritten notes were posted on the walls to show the CE Studio participants that their comments were valued and captured for consideration. This emphasized the transparency of the research team and increased trust from the participants. A master transcript of the session, merging all the recordings, was developed for the researchers to consider changes to their study, survey, and all elements of the research. An audio recording was kept as a backup to the master transcript.

A follow‐up email was sent to the CE Studio participants to thank them for their time and for sharing their experiences and expertise. They were invited to reach out to the research team for extra information or guidance on the study, survey, and research elements. This was done so that the participants who may not have felt that they could speak in a group had a way of communicating anything that they felt was necessary.

### 2.4 Post Community Engagement Studio

After the CE Studio session, the research team reviewed the session notes and recordings collected during the discussion. Themes, context, wording, and presentation of the survey were re‐evaluated based on the feedback received, and the survey was adapted accordingly.

## 3 Results

Table 2 presents the main themes generated in response to the questions asked during the CE Studio session, and Table 3 highlights some of the main changes made to the survey after the session. Participants made valuable contributions. Overall, 130 edits were made after receiving feedback from the community experts. The edits included changes in both the wording and the context, tailoring the questions to the purpose of the research and addressing the concerns pointed out by the community experts. One suggested edit to motivate participation was to highlight the purpose of the study and how the survey results would be used. Therefore, the research team developed a brief introduction using plain language to explain the need for the survey and how the information collected would benefit the community in the future.

**TABLE 2:** Discussion questions, prompts, and corresponding feedback from a Community Engagement Studio.

Topic	Discussion questions or prompts by the facilitator	Additional prompts (ice breakers)	Feedback
Dental access experience	What is your child's dental care like?	What is it like to take them to the dentist?	Challenging
		What may be your frustration as a parent?	Face some barriers, especially with children with special needs and juggling work and school time.
Barriers	What are your personal barriers to accessing dental care for your adolescents?; Why do adolescents not follow the treatment plan?; Why would you not show up to an appointment?; How does your adolescent view their own dental hygiene?	Question from the panel: Do you get patients who like to get treated (shots?)	Generational and family circumstances; relate the treatment with pain and fear; rudeness; cost; no reminders; do not take dental hygiene seriously
	What would you want to know before appointment?; What may be your frustration as a parent?; What are we missing?		Number of visits; sequencing; transportation; commute time; cost; lack of information; community engagement; know their needs; education; communication
Overall Survey	Do the questions make sense?; Any other categories that don't make sense?	Do you want the instructions to clarify that it is not a test?; Is it important to have I don't know or not reported?	Streamline instructions and state it is not a test. Not a problem to say I don't know the answer. Demographics, marital status, and income level. Add “I don't know or not reported.”
Questionnaires on different topics	Do you think we need to change the format?	Multiple choice or fill in the blank?; What about the instructions?; How do you feel about the pictures and labels?	Multiple choices, streamline instructions and be larger and in color.
Conclusion	Would you do this survey?		Compensation is important.
	How long do you think it would take to complete?; Is the survey accessible?	Would you take this survey digitally?	10–15 min; yes, but internet access may be a barrier
	What barriers should we consider with this survey?		Time compensation
	Does anything else make them want to participate or not?		Friendly wording and format

**TABLE 3:** Survey main changes after Community Engagement Studio.

	Expectation	Change
First approach	Being informed about the reasoning of the questions would motivate people to engage and complete the survey	An interest form with a brief explanation about the survey and why the questions were important to develop better strategies for dental care access among populations with health disparities
Demographics	Make the survey even more inclusive; make the participants feel comfortable about their status; avoid any judgmental feeling	“Choose all that apply” was included in questions about the parent/caregiver's race as well as the child's race; “I do not know and/or I don't want to answer” were included in the multiple‐choice options; simplified the multiple‐choice options about employee status and education
Oral health status, access, and barriers	The previous format included 19 questions. The final product has 11 questions.	Smaller number of questions and more straight to the point
Oral health literacy	Multiple choices; interactive and informative	Less and better‐quality images; less questions targeting minimum knowledge of oral anatomy, health/disease recognition, and dental appointment information
Modified Dental Anxiety Scale	This form was very well received by the audience	No changes in the wording or format. Added to the adolescent section of the survey to have their insights on dental care experience
Dental care satisfaction	Effective in addressing what really matters for the individual seen by a dental provider; and save time	Made sure topics such as access, cost, services, care from dentists and staff, and patient's ratings were addressed; branching logic was added according to two different situations: (1) If the child has dental insurance; (2) If the child has a regular dentist. If the answer was “no,” the respondent would have access to less questions and more generic ones such as: “The fees dentists charge are too high: YES/NO”; Detailed questions about dental insurance plans and/or dental appointment experiences with a regular dentist were kept only for those participants that answer “YES.”
Health Literacy Assessment Tool	Make it more accessible for kids aged 12–17 years old	The wording of this instrument was adjusted for 4th graders

Some CE Studio participants questioned the need for standard demographic information, such as race, ethnicity, marital status, and income level. The participants noted some discomfort with having to disclose this information. After explaining that some of the health disparities observed among certain groups could be explained by these factors, they were more open to the inclusion of these questions.

When we asked the community experts about competing priorities impacting their dental health, they mentioned that housing, lack of grocery stores, and food insecurity were more important priorities than access to dental care. Some of the community experts stated that oral health was not a priority. In response, we added an option to the questions about barriers to dental care, allowing upcoming research subjects to indicate that visiting dental providers was not a priority for them. Interestingly, the community experts also mentioned that they struggled with access to dental care during their childhoods, highlighting a generational need for dental care and the need to bring oral health awareness to this community.

Anxiety and fear before and during dental appointments are barriers to dental care, and empathy from the dental provider and staff is essential to help patients overcome them. The Modified Dental Anxiety Scale (MDAS) [21] was already part of the survey. However, we added the Consumer Assessment of Healthcare Providers and Systems (CAHPS) [27] asking about the patient's experience and provider's empathy during the dental appointment.

Although the survey seemed long when printed on paper, the CE Studio participants pointed out that they would need 15–20 min to complete it and that compensation would encourage completion. They suggested several changes to the format to make it feel more like a survey than a test. Participants emphasized that the survey was user‐friendly, making it easy to select answers and requested images to make the questions more self‐explanatory. Taking this into consideration, we worked alongside the UB REDCap core to create a better layout and flow across the different themes and questions, making it even more fluid and accessible.

Even though the community experts liked the idea of completing the survey online, the fact that Internet connection would be a limitation for many people from their neighborhood was highlighted. Thus, the research team will offer to meet upcoming research subjects at the community center to give them access to portable electronic devices and stable Internet access to complete the survey developed after the CE Studio, as part of the associated study. The research team will also be prepared to offer remote assistance such as step‐by‐step instructions over the phone, text, or email.

## 4 Discussion

The CE Studio proved to be effective in dental service research, providing valuable feedback that enhanced the survey's clarity, relevance, and accessibility before its use in an upcoming study on barriers to dental care access among adolescents. All qualities were emphasized by the facilitators and community experts present at the CE Studio. As suggested during the session to motivate participation, the survey should clearly highlight the purpose of the study, and how the results would be used. This demonstrates the need to provide clear information at the beginning of the survey, which may help address the challenges faced by community members face when engaging in research [31, 33]. Additionally, the CE Studio's feedback emphasized the importance of listening directly to community members about their lived experiences, perceptions, and concerns [32, 33, 37]. Studies have shown that engaging the community early and throughout the research process can accelerate the translation of findings into practice and increase the likelihood that those findings will be embraced by the population of interest [43, 44].

Participants in the CE Studio also stated that factors such as housing, food insecurity, and a lack of grocery stores were more pressing concerns than access to dental care. This feedback led to the inclusion of response options that reflected these competing priorities. This highlights the importance of community‐engaged studies to inform research in a real‐world context, especially regarding how social and structural systems shape health behaviors [29, 45].

There are few examples in the literature of research strategies designed to engage community members experiencing health disparities [31, 34, 36, 46]. However, some studies have emphasized that community participation is hindered by lack of recognition of their perspectives and needs [47, 48, 49]. Thus, incorporating community engagement into the design of research on access to dental care is crucial, as this topic is influenced by different factor levels (e.g., at the individual, interpersonal, institutional, community, and political levels). A multifactorial approach is necessary, and the CE Studio ensured that the survey design considered both the investigator's perspective and that of the community. Moreover, the process empowered community experts by deepening their understanding of the research process and fostering connections with fellow community members. Through these interactions, they may have learned to appreciate the value of research and the importance of collaboration between researchers and the community [41, 44], which represents a potential secondary benefit to this approach.

While CE Studios provide a more comprehensive context for healthcare research development tools and discussions with communities, they have some limitations. As with any community‐engaged project, the voices represented in the CE Studio may not be representative of the entire community. It is important to ensure the involvement of a diverse group of community experts to provide a broad range of perspectives to support the study design and questions posed by the researchers. Feedback obtained from CE Studios is mostly narrative and may have limitations depending on the associated research question. Additionally, CE Studios incur costs and depend on academic institution funds for adequate implementation. If the academic institution does not offer the infrastructure, staff, and funds to adequately implement this community engagement model, it is recommended to plan the CE Studio in advance and include it as part of the grant budget.

Despite these limitations, this experience provided our research team with the opportunity to refine the survey, including its format, recruitment materials, and consent document, before initiating the study on barriers to dental care access among adolescents. The protocol for administering the revised survey was also adjusted. After receiving approval for all survey revisions from the UB IRB and setting up the compensation method, the research team will start recruiting and enrolling participants to pilot test the survey on access to dental care. Following the pilot phase, the team will initiate community outreach efforts to actively recruit and enroll additional participants, ensuring that the community's perspectives and needs are fully incorporated into the research.

## 5 Conclusions

Although CE Studios have been used to inform different types of research, using this approach to inform health behavior research in the field of dental services is innovative and promising. Individual participation and community involvement are necessary to successfully increase the quality and relevance of research and translate results, aligned with community needs, into practice. The findings of this CE Studio underscore the invaluable contributions of community experts, both to the community and to preventive dentistry. They bring first‐hand experience as members of the intended population, and they also serve as ambassadors for further engagement within their communities. By sharing their experiences and encouraging others to participate in the research, they may enhance the overall trust and confidence in the research process. By leveraging the insights and experiences of community experts, researchers can address health disparities and develop more effective interventions.

## Conflicts of Interest

The authors declare no conflicts of interest.

## Data Availability

The data that support the findings of this study are available on request from the corresponding author. The data are not publicly available due to privacy or ethical restrictions.
